# The Impact of VR-CALM Intervention Based on VR on Psychological Distress and Symptom Management in Breast Cancer Survivors

**DOI:** 10.1155/2022/1012813

**Published:** 2022-06-07

**Authors:** Xiuqing Zhang, Senbang Yao, Menglian Wang, Xiangxiang Yin, Ziran Bi, Yanyan Jing, Huaidong Cheng

**Affiliations:** ^1^Department of Oncology, The Second Affiliated Hospital of Anhui Medical University, Hefei, Anhui, China; ^2^Department of Oncology, Anhui Medical University, Hefei, Anhui, China

## Abstract

**Objective:**

To evaluate the effectiveness and feasibility of Managing Cancer and Living Meaningfully based on VR (VR-CALM), which is used to manage expected symptoms of cancer itself, relieve psychological distress, and improve quality of life (QOL) in the Chinese breast cancer survivors (BCs).

**Methods:**

Ninety-eight patients with breast cancer were recruited in this study. These patients were randomly assigned to the VR-CALM group or the care as usual (CAU) group. All patients were evaluated by the Functional Assessment of Cancer Therapy-Breast cancer patient (FACT-B), Distress Thermometer (DT), Concerns About Recurrence Scale (CARS), Piper Fatigue Scale (PFS), Pittsburgh Sleep Quality Index (PSQI), The Self-Rating Anxiety Scale (SAS), and The Self-Rating Depression Scale (SDS) before and after VR-CALM or CAU application to BCs. We compared the differences in all these scores between the VR-CALM group and the control group.

**Results:**

Patients in the VR-CALM group showed a significant decrease in levels of distress, anxiety, depression, sleep disorders, and fatigue (*t* = −6.829, *t* = −5.819, *t* = −2.094, *t* = −3.031, *t* = −10.082, *P* ≤ 0.001, 0.001, 0.05, 0.01, 0.001, respectively) and had higher level of quality of life (*t* = 8.216, *P* ≤ 0.001) compared with the CAU group after intervention. And postintervention patients in VR-CALM group compared with preintervention showed lower level of distress and remarkable improvement of QOL (*t* = 11.521, *t* = −10.379, *P* ≤ 0.001, 0.001). The preintervention questionnaire revealed no significant between-group differences regarding distress, anxiety, depression, sleep disorders, fatigue, and quality of life.

**Conclusion:**

VR-CALM is a psychotherapy tailored to the needs of patients with breast cancer. This research innovatively used VR-based CALM intervention to improve psychological and chronic symptoms in BCs. The results of the present study indicate that VR-CALM has salutary effects on the improvement of QOL and relieves psychological distress, anxiety, depression, sleep disorders, and fatigue in BCs.

## 1. Introduction

People with cancer are living longer than they did in past, especially breast cancer survivors (BCs). At present, the five-year survival rate of breast cancer is as high as 90%, far higher than that of other cancers, indicating that cancer has been transformed into a chronic disease [1]. While in 2020, breast cancer in women surpassed lung cancer for the first time as the most common cancer worldwide, accounting for approximately 11.7 percent of new cancer cases according to the global cancer observatory, which is due to the improvement of breast cancer diagnosis and treatment, as well as BCs' overall process management. Since 1999, the NCCN has provided updated guidelines for the management of psychological distress in cancer [2]. According to studies, around one-third of cancer patients have psychological discomfort, and clinically significant psychological symptoms have been found in 38% of cancer survivors [3, 4]. Most cancer patients experience symptoms, and their incidence and severity vary by cancer type, stage, treatment, and comorbidities [5, 6]. Symptoms can be caused by cancer itself, early or late therapeutic side effects, and comorbid conditions, and simultaneously symptom management is critical for treatment effectiveness, necessitating the development of innovative approaches by healthcare professionals to improve quality of life [7].

Gary Rodin suggested that CALM has a positive effect on symptom management in patients with advanced cancer, whereas CALM is designed to help patients live with cancer and decrease psychological distress [8]. CALM is comprised of four major components: (1) symptom control and communication with health care providers; (2) changes in self and relationships with others; (3) spiritual well-being and the meaning of life; (4) communicating about future concerns, hopes, and mortality [9]. One small sample randomized controlled experiment has demonstrated the effectiveness of CALM intervention in the improvement of cognitive impairment and QOL and relieving psychological distress in breast cancer patients [10]. A study demonstrates the feasibility and treatment potential of VR-CBT in patients with generalized SAD and its results suggest that VR-CBT may be effective in reducing anxiety as well as depression and can improve quality of life [11]. This study was conducted through virtual reality (VR) technology, which immerses the patients in a computer-generated virtual environment. It is a head-mounted device that projects a virtual picture as well as noise-cancelling audio. The advancement of VR software allows for increased involvement and greater immersion [12]. Our research team's original VR scenarios include a beach house and Butterfly Valley, with original lead phrases. One study showed that high-interactive VR systems are more effective [13]. Therefore, interactivity and immersion are key factors affecting VR efficacy.

VR-CALM intervention is to apply VR technology to the intervention process of CALM. Traditional CALM intervention mode is based on the communication between CALM therapists and patients. VR-CALM is to immerse patients in virtual reality in beautiful environment like Butterfly Valley and seaside, while listening to ambient sounds as well as instructions provided by the CALM therapist. The instructions follow CALM intervention manual and offer intervention in four areas, including symptom management and health guidance, analysis of how illness has changed people and their relationships with those close to them, exploration of meaning and purpose in life, and talk about the future and hope. And at the end of each session, there will be a special time and place to give patients an opportunity to “speak,” where they can tell what is on their mind, why they are unhappy, and patients are provided with specific guidance about their specific problems.

Since the effectiveness of CALM intervention and VR-CBT has already been proven, there is no study discussed whether VR-CALM can manage expected symptoms of breast cancer itself. In this context, we undertake this randomized controlled experiment to explore the availability of VR-CALM intervention relative to care as usual in both symptom management and psychological distress including quality of life, anxiety, depression, distress, concerns about recurrence, fatigue, and somnipathy.

## 2. Materials and Methods

### 2.1. Design

This is a nonblind, parallel assignment randomized controlled trial (RCT).

### 2.2. Sample

A total of 98 breast cancer patients receiving at least 2 courses of regular chemotherapy was recruited from the Second Affiliated Hospital of Anhui Medical University between January 2021 and August 2021.

### 2.3. Randomization

The statistical staff of our team, who did not involve in the experiment, managed the randomization. After participants' baseline assessments, statistical staff provided computer-generated random assignments. The researchers were unknown about the sequence, which was written on a card, sealed in an envelope, and opened when dispensed.

### 2.4. Inclusion and Exclusion Criteria

Inclusion criteria were as follows: (1) pathologically diagnosed breast cancer patients, completion of at least 2 cycles of regular chemotherapy, with no intolerable side effects; (2) Karnofsky performance status score ≥80; (3) no auditory, visual, language, and other functional disorders; and (4) age from 18 to 70, with a sufficient ability to complete the necessary tests for the study. Exclusion criteria were as follows: (1) patients with advanced cachexia; (2) patients with obvious anxiety, depression, and other mental symptoms; (3) lack of adequate baseline bone marrow and organ reserves or associated with serious heart, liver, kidney, brain, and hematopoietic diseases; (4) patients with brain metastases and other brain diseases; and (5) patients with a history of alcohol or drug dependence and taking cognitive-improving medications.

### 2.5. Procedure

BCs were identified through prescreening of test results, and eligible patients were recruited during hospital stays in the oncology department and breast surgery department. Oncologists presented patients with experiments and VR-CALM intervention methods and obtained informed consent from the patients and their families. The researchers next assessed whether the patients meet the requirement and performed baseline measurements. Then, statisticians randomly divided the participants into two groups. The intervention was conducted in an oncology conference to ensure patient privacy during hospitalization. After six cycles of intervention, complete follow-up assessments were conducted during patients' hospitalization or by telephone.

### 2.6. Measures

#### 2.6.1. QoL

Quality of life was assessed by the Functional Assessment of Cancer Therapy-Breast cancer patient (FACT-B). Quality of life scores were recorded at both baselines and after 6 cycles of VR-CALM intervention. This study used the fourth vision of FACT-B that includes four domains: physical well-being, social/family well-being, emotional well-being, and functional well-being, and a subscale for BCs (rated by 28, 28, 24, 36, and 20 points, respectively) and has better reliability and validity. The higher the total score, the better is the QoL.

#### 2.6.2. Distress

The Distress Thermometer (DT) was developed as a Distress Management Screening Measure (DMSM) for assessing the level of distress and was recommended by the National Comprehensive Cancer Center. It is a visual analog scale ranging from 0 (no distress) to 10 (extreme distress). Patients with a score above 4 are considered psychologically distressed.

#### 2.6.3. Anxiety and Depression

The Self-Rating Anxiety Scale (SAS) is a norm-referenced scale used as a screener for anxiety disorders with 20 items. Patients with a score of 40 or more illustrate anxiety. The higher the total score, the higher the degree of anxiety. The main indicator of the Self-Rating Depression Scale (SDS) is the total score, and the cutoff point for depression is 50 points. A higher score indicates a higher degree of depression.

#### 2.6.4. Concerns about Recurrence

Concerns About Recurrence Scale (CARS) consists of two domains: the overall Fear of Cancer Recurrence (FCR) and the nature of women's FCR. The first part contains 4 items: measure the perceived likelihood of a recurrence of cancer; how often participants thought about a recurrence; how long they thought about a possible recurrence; and how emotionally painful thoughts about a recurrence were [14]. The second section measures the nature of women's concerns about recurrence. The higher the total score is, the more significant the FCR tendency is.

#### 2.6.5. Fatigue

Piper Fatigue Scale (PFS) is the most effective instrument to assess the perceived fatigue of patients with chronic disease, especially cancer, with 24 items and each item has 11 response categories from 0 to 10. The severity of fatigue represented by each score is as follows: 0 = no fatigue, 1–3 = mild fatigue, 4–6 = moderate fatigue, and 9–10 = severe fatigue. The greater total scores suggested a higher degree of fatigue.

#### 2.6.6. Somnipathy

Pittsburgh Sleep Quality Index (PSQI) is the most commonly used universal measurement method in both clinical and research. The PSQI was developed traced back to 1988, with no aimed population [15]. PSQI includes 7 parts: the quality of sleep, sleep time, the amount of sleep, sleep efficiency, somnipathy, the hypnotic drug, and the daytime dysfunction. Previous studies have shown that each part score measured a particular aspect of the construct of sleep quality [16]. The final score is the sum of seven partial scores and higher scores indicate poorer sleep quality.

### 2.7. Intervention

VR-CALM intervention was conducted by 1 psychologist, 1 oncologist, and 3 postgraduates with the certificate of psychological consultant from the Department of Oncology, the Second Affiliated Hospital of Anhui Medical University. All VR-CALM therapists received relevant professional training and ongoing supervision from clinical researchers and were qualified to provide VR-CALM intervention to patients. The group supervision meeting was held once a week for case discussion and the summary in order to ensure that the VR-CALM intervention and the trail going on wheels. VR-CALM is a brief, manual form of personal psychotherapy [9, 17]. The intervention course for VR-CALM was 3 months, during which patients in the intervention group would receive 6 separate treatments for VR-CALM, each time for 30 min, and the first 3 treatments would be completed in the first month. The VR equipment consisted of a head-mounted glasses and two controllers. The patients wearing the equipment will find themself immersed in a beauty spot such as a seaside and Butterfly Valley and are allowed to walk around. During this process, patients can hear wind and intermittent guidance. And patients are able to touch butterflies with the controller in their hands. This immersive experience of audio-visual integration constitutes a complete intervention model. Each patient will experience these scenes at least 2 times, and then they can choose which scenery they prefer for the next intervention. This also ensures the consistency of intervention for each patient.

### 2.8. Statistical Analysis

All the data were collected and analysed by Statistical Package for the Social Sciences (SPSS, V22.0) and were expressed as the mean ± SD. The Chi-squared test was used in the comparison of classification data, and the paired *t*-test was used in the comparison before and after the same group. The unpaired *t*-test was used to compare the differences between different groups. The data differences were determined as statistically significant at *P* < 0.05.

## 3. Results

### 3.1. The Baseline Characteristics of the Research Enrolled Patients and Flow

**Figure 1:**
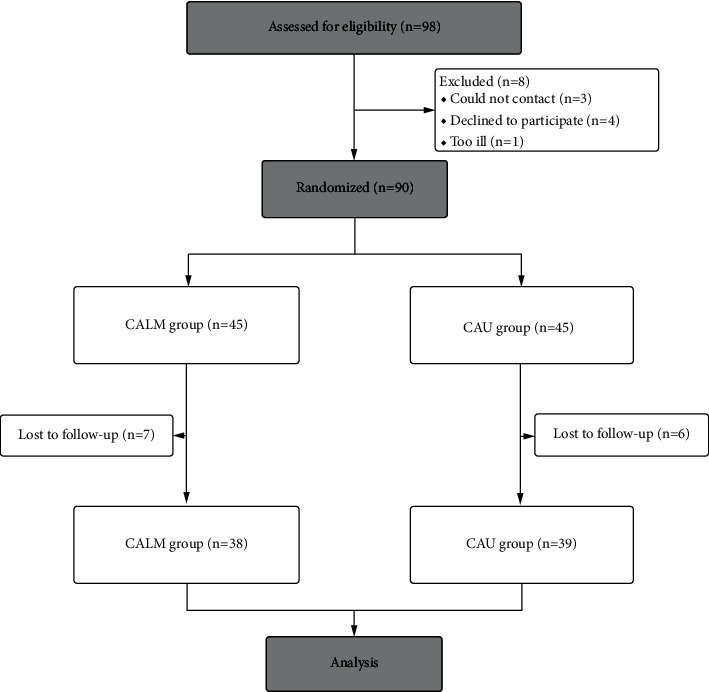
Consort flow diagram.

Various patients' demographics, including age, education, surgical method, and postoperative pathological information, were obtained at the time of enrolment (Table 1). The results suggest that there were no significant differences in demographic information, including age (*t* = 0.708, *P*=0.481), years of education (*t* = −1.182, *P*=0.241), clinical information including the Karnofsky performance status (*χ* = 0.317, *P*=0.573), and the tumour stage (*χ* = 0.445, *P*=0.247, *P*=0.961) between the VR-CALM group and CAU group. The flow diagram showed that 98 breast cancer patients are eligible to take part in the study between January 2021 and August 2021 (Figure 1), 90 of whom were randomly assigned to VR-CALM (*n* = 45) and CAU (*n* = 45). However, 7 people did not complete the VR-CALM intervention and 6 people in the CAU group did not complete the final assessment. Finally, there were 38 people in the VR-CALM group and 39 in the CAU group.

### 3.2. Comparison of the Symptom Assessment before and after Intervention Periods within Groups

The performance of the BCs on the Anxiety and Depression test, Concerns About Recurrence Test, Fatigue test, somnipathy test, DT, and QOL evaluation scale before and after VR-CALM or CAU are shown in Table 2. Compared with the CAU group, the performance of the VR-CALM group had remarkable changes in the overall scores of the FACT-B (*t* = −10.379, *P* ≤ 0.001), SAS (*t* = 4.680, *P* ≤ 0.001), SDS (*t* = 4.101, *P* ≤ 0.001), CARS (*t* = 2.742, *P* ≤ 0.001), PFS (*t* = 9.913, *P* ≤ 0.001), and PSQI (*t* = 6.066, *P* ≤ 0.001) before and after VR-CALM, but for the CAU group, there were substantial variations in total scores on the FACT-B (−2.988, *P* ≤ 0.01), SAS (*t* = 22124.817, *P* ≤ 0.001), CARS (*t* = 2.370, *P* ≤ 0.05), and DT (*t* = 3.328, *P* ≤ 0.01) before and after CAU, while no significant differences were found in SDS (*t* = −1.517, *P*=0.138), PSQI (*t* = −1.839, *P*=0.074), and PFS (*t* = 1.515, *P*=0.138). When these data were combined, the magnitude of data changes in the VR-CALM group was higher than in the CAU group, despite the fact that both groups' performance demonstrated significant change.

### 3.3. Comparison of the Symptom Assessment before and after Intervention Periods between Groups

The differences between the two groups before and after VR-CALM or CAU are shown in Table 3. At the start of the study, there were no statistically significant differences between the VR-CALM group and the CAU group on any of the scale scores: FACT-B (*t* = 1.411, *P*=0.162), DT (*t* = −1.806, *P*=0.076), SAS (*t* = 1.105, *P*=0.273), SDS (*t* = 1.315, *P*=0.194), CARS (*t* = 1.869, *P*=0.068), PFS (*t* = 0.687, *P*=0.494), and PSQI (*t* = 0.506, *P*=0.614), which means that there was no significant difference between the two groups at the baseline before the VR-CALM or CAU. However, 3 months after the interventions, the two groups showed statistically significant differences: FACT-B (*t* = 8.216, *P* ≤ 0.001), DT (*t* = −6.829, *P* ≤ 0.001), SAS (*t* = −5.819, *P* ≤ 0.001), SDS (*t* = −2.094, *P* ≤ 0.05), CARS (*t* = 1.170, *P*=0.247), PFS (*t* = −10.082, *P* ≤ 0.001), and PSQI (*t* = −3.031, *P* ≤ 0.01).

## 4. Discussion

The purpose of this study is to evaluate the effectiveness of VR-CALM intervention in symptom management and psychological distress relief in BCs. Our study found that VR-CALM intervention was more effective than CAU in most of the variables (QOL, psychological distress, anxiety, depression, sleep disorders, and fatigue). These findings are consistent with the previous studies [8, 17–22]. It is highlighted that psychological care is beneficial for patients with advanced cancer according to international machinery such as the European Palliative Care Research Collaborative (EPCRC), the Worldwide Palliative Care Alliance, the World Health Organization, and the International Psycho-Oncology Society. While VR-CALM is a psychological intervention tailored to each individual patient to help prevent and treat adverse psychological reactions, improve quality of life, and give meaning to life.

The CALM intervention has proven to be a useful treatment, providing a systematic approach to alleviate the suffering in cancer patients [8]. CALM builds on a decade of theoretical and empirical work and focuses on the influence of many factors, including the severity of physical symptoms, attachment style, and other interpersonal variables [23]. CALM is conducive to address practical and existential problems faced by cancer patients [9], including symptom management, role change, and mental-, existential-, and death-related issues. According to the CALM therapy manual, the components that CALM contributes to its therapeutic effectiveness are the creation of meaning, the renegotiation of attachment security, the regulation of emotion, and mentalization, all within a true supportive relationship [24].

CALM is unique as it is based on relationship, attachment, and existentialist theories and specifically addresses the specific issues of advanced cancer. Preliminary results [14, 25] and results from a large randomized controlled trial in Toronto [26] are encouraging, suggesting that CALM is a promising intervention for patients with advanced or metastatic cancer. Because culture shapes cognitive, emotional and behavioural responses to cancer and cancer treatment, awareness and knowledge of treatment options, and acceptance of psychological interventions [27–29], the use of specific interventions (e.g., CALM) needs to be examined in a specific cultural context. Effective mechanisms may include offer the patients, doctors, nurses and other health care providers the opportunity to communicate, solve the impact of disease to their self-awareness and family relationships, find or regain the meaning of life and goals, express and manage the fear and desire related to the end of the life, and begin to prepare for the end of life [26]. Previous evidence has indicated that there are changes in cytokine levels in BCs with cognitive impairment, and a correlation was noted between cognition and QOL in BCs [30]. It may be the underlying mechanism of CALM intervention to improve QOL.

Many studies and clinical trials use VR as a simulation, interactive, and distraction tool for patients with mental disorders such as posttraumatic stress disorder (PTSD), anxiety, specific phobias, schizophrenia, autism, dementia, and severe stress [31]. A study has shown that VR-based interventions are particularly important when compared to traditional cancer symptom management interventions [32]. First, VR-based cognitive training allows cancer patients to learn [32]. VR-based interventions provide immediate feedback on patient performance and can be adapted to suit patient needs [33]. In addition, VR-based interventions combine the latest real-time graphics and imaging technologies to allow patients to experience a multitude of visual and auditory stimuli in a computer-generated virtual environment to meet their rehabilitation needs [34, 35]. A previous study has shown that distracting interventions can effectively alleviate the adverse reactions and symptoms in cancer patients undergoing chemotherapy [36]. Among these interventions, virtual reality, with its powerful attention-grabbing power, has proved effective for cancer patients in different settings [37]. VR allows patients to temporarily break away from the medical environment and immerse themselves in a pleasant atmosphere, thus producing pleasant emotions and reducing negative emotions [12, 38]. Previous literature has indicated that VR is a useful intervention for relieving anxiety, depression, pain, and fatigue during chemotherapy [32, 39–44]. But there is no study that has proven its effectiveness on symptoms caused by cancer itself, which lasts longer and are more destructive than symptoms related to chemotherapy. Nevertheless, discomfort caused by wearing a VR device including headaches, eyestrain, and nausea is also a confounding factor that cannot be kicked out [45].

Psychological symptoms should be synthetically assessed in all patients. In the latest NCCN guidelines, experts suggest that a complete symptom management should provide psychological and emotional support to both the cancer patients and their family caregivers.

Distress is a multifactorial unpleasant experience and may have a negative impact on patients' ability to cope with cancer, symptom, and treatment. The distress should be recognized, monitored, documented, and treated promptly at all the stages and in all the circumstances [2]. In addition, patients should be assessed for psychological distress at each visit ideally, at least during the first visit or at appropriate intervals [2]. The present study found that VR-CALM is effective for relieving distress in BCs, and CAU group also had a remission after intervention, while there was still a difference compared with the VR-CALM group.

Previous studies estimated that 10% of cancer survivors live with anxiety and 16% have a major depressive disorder [46]. The study by Madhusudhana et al. confirmed that anxiety and depression are associated with longer treatment initiation intervals [47]. So, it is necessary for oncologists to intervene with this anxiety and depression. Our finding confirmed the usefulness of VR-CALM in reducing BCs' anxiety and depression.

Fear of cancer recurrence (FCR) is a concept of fear, worry, or concern about cancer returning or progressing [48]. The majority of BCs are concerned about possible disease progression and fear of cancer recurrence [49]. The estimated prevalence of moderate to high levels of CAR ranges from 24% to 56%, while FCR is one of the most common chronic and severe problems for cancer patients [50]. A systematic review shows that most BCs have problems in dealing with FCR which needs specialists' help [51]. And Cognitive Behavioural Therapy (CBT) is a common treatment for FCR as its effectiveness has already been proven in previous studies [1, 52–54]. This study did not find any evidence that VR-CALM has influence on easing fear of cancer recurrence in BCs, as well as CAU.

Fatigue is a subjective and unpleasant symptom, which is a general feeling that interferes with normal work and life [55]. Previous studies have shown that one-third of breast cancer patients have persistent sleep disturbances [56]. However, sleep disorders are associated with a greater fatality risk in BCs [57, 58]. A randomized trial revealed that compared with usual care, the physical activity intervention would improve sleep quality in self-report [59]. The present study revealed that both VR-CALM and CAU are valid in decreasing fatigue, while the effect of VR-CALM intervention is more obvious.

Quality of life assessment is an important criterion for women recuperating from cancer [60, 61]. Ferguson et al. research suggested that CBT can compensate for cognitive deficits and improve QOL [62]. While medications such as methylphenidate, modafinil, and epoetin may improve cognition by reducing fatigue and boosting sobriety, its effectiveness has not been proven in previous studies [63]. It advocates regular physical exercises as effective tools for relieving fatigue, stress, and depression and quality of life in BCs [64–66]. The present study found that after VR-CALM intervention, BCs in the VR-CALM group had a better quality of life than those in the CAU group.

Breast cancer patients who participated in VR-CALM reported that VR-CALM provided an opportunity for them to get out of the ward and out of the house and see the outside world. VR-CALM provided a time and place for patient-to-patient communication as well as opportunities for further communication with doctors and nurses. In addition to discussing, disturbing, and frightening issues such as treatment and prognosis of diseases, family communication has been enhanced and family experience sharing has been facilitated. The primary goal of breast cancer patients is to address the issues that are troubling them in life and the survival and psychological issues that arise after a cancer diagnosis, highlighting the importance of the safe space provided by the VR-CALM intervention. In addition, although cancer patients feel loved and cared for by their families, they also want to alleviate the pain caused by their disease, which can lead to further anxiety and depression due to family and financial burden. Patients in the VR-CALM intervention group reported after the intervention that the VR immersive experience provided them with emotional relief and relaxation, and this was considered an achievable goal in this population. In addition, the importance of CALM therapists was highlighted in the course of this study. CALM therapists have nonjudgmental compassion and an emphasis on reflection. Patients indicated that they valued the therapist's point of view as a professional and that CALM therapist's point of view helped solve the problem and made patients feel that someone understood their situation and experience. It also suggests that oncology professionals who are trained in psychotherapy and have compassion and understanding for patients can offer CALM interventions. In addition, the study found that the advantage of the CALM intervention is that oncologists can be trained to provide CALM as long as the integrity of the treatment is maintained through continuous monitoring. Although there may be subject-specific differences in oncological knowledge among therapists, there is certainly much in common and CALM interventions are not fundamentally different based on the expertise of the providers.

In general, psychological intervention superior to medications in terms of symptom management and improving quality of life, with no adverse effects. Therefore, the psychological intervention has become the most effective and appropriate strategy for symptom management, which also promotes the conduct of this research. The current review focused on the impact of VR and CALM interventions. These therapies have been used in a variety of medical and psychological sectors, yielding findings that, if validated by more rigorous trials, might have a considerable impact on healthcare. However, the success of integrating VR and CALM interventions in ordinary clinical practice will also be determined by their practical viability. Compared with similar studies, this research innovatively used VR-based CALM intervention to improve psychological and chronic symptoms in breast cancer patients. The findings provide a simple and easy-to-adopt intervention and will help to better help breast cancer patients recover their mental health.

Some design limitations merit further comments. First, the small sample size does not allow comparisons between subgroups of patients to determine moderators of treatment outcome and may not represent the actual difference in improvement between the CAU group and the VR-CALM group. And this study did not explain the specific mechanism of symptom management of VR-CALM. Secondly, the short follow-up period of this study could not prove the long-term effectiveness of VR-CALM intervention.

## 5. Conclusion

VR-CALM intervention strongly reduced psychological distress and improved quality of life among breast cancer survivors in daily life. In the 8-month follow-up, patients who participated in VR-CALM also reported to have less distress, anxiety, depression, and sleep disorders. The study did not find robust evidence that both VR-CALM and CAU can ease the patient's fear of cancer recurrence. Our finding suggests the potential viability of VR as a new type of CLAM intervention approach to relieve psychological distress and improve quality of life in BCs. Furthermore, if future research is agreeing to this study about the usefulness of VR-CALM in symptom management in BCs, this could promote the research of CLAM mechanism.

## Figures and Tables

**Table 1:** Comparison of demographic characteristics and clinical data of breast cancer between the VR-CALM group and the CAU group.

	VR-CALM	CAU	*t*/*χ*	*P*
Age (years)	52.29 (7.686)	51.03 (7.979)	0.708	0.481
Education (years)	8.55 (2.435)	9.21 (2.408)	−1.182	0.241
Surgical method			0.112	0.738
Breast conservative surgery	3	5		
Mastectomy	35	34		

*Tumour stage*				
I	6	7	0.445	0.961
II	16	18		
III	11	10		
IV	5	4		

*Molecular classification*			1.321	0.724
Luminal A	4	6		
Luminal B	17	20		
HER-2 overexpression	12	10		
TNBC	5	3		

*Pathological type*			1.021	0.692
Invasive carcinoma no special type	34	33		
Invasive carcinoma special type	1	3		
Noninvasive carcinoma	3	3		

*KPS*			0.317	0.573
80	14	12		
90	24	27		

Data are presented as the mean ± SD. Abbreviations: SD, standard deviation; KPS, Karnofsky performance status; VR-CALM, Managing Cancer and Living Meaningfully based on VR; CAU, care as usual.

**Table 2:** Separate comparison of symptoms in the 2 groups of breast cancer patients before and after VR-CALM or CAU.

Group	*N*	FACT-B	DT	SAS	SDS	CARS	PFS	PSQI
VR-CALM group								
BCT	38	78.74 ± 10.125	5.71 ± 1.250	51.66 ± 11.252	51.32 ± 11.552	59.34 ± 13.581	103.03 ± 8.645	10.45 ± 3.438
ACT	38	100.47 ± 14.33	2.95 ± 1.845	44.16 ± 11.083	46.63 ± 9.824	55.82 ± 10.557	77.79 ± 11.548	8.74 ± 2.565
*t*		−10.379	11.521	4.680	4.101	2.742	9.913	6.066
*p*		≤0.001	≤0.001	≤0.001	≤0.001	≤0.01	≤0.001	≤0.001
CAU group								
BCT	39	75.44 ± 10.39	6.13 ± 0.695	49.31 ± 6.814	48.64 ± 4.934	55.00 ± 4.605	101.62 ± 9.35	10.05 ± 3.426
ACT	39	79.44 ± 6.703	5.49 ± 1.393	55.21 ± 3.806	50.21 ± 3.806	53.49 ± 6.328	99.77 ± 6.964	11.26 ± 4.494
*t*		−2.988	3.328	−4.817	−1.517	2.370	1.515	−1.839
*p*		≤0.01	≤0.01	≤0.001	0.138	≤0.05	0.138	0.074

*Note.* FACT-B, Functional Assessment of Cancer Therapy-Breast cancer patient; DT, Distress Thermometer; SAS, The Self-Rating Anxiety Scale; SDS, The Self-Rating Depression Scale; CARS, Concerns About Recurrence Scale; PFS, Piper Fatigue Scale; PSQI, Pittsburgh Sleep Quality Index; BCT, before CALM/CAU treatment; ACT, after CALM/CAU treatment; SD, standard deviation. Data are presented as mean ± SD.

**Table 3:** Comparison of symptoms between the VR-CALM group and the CAU group of breast cancer patients before and after VR-CALM or CAU.

Group	*N*	FACT-B	DT	SAS	SDS	CARS	PFS	PSQI
Before VR-CALM or CAU								
VR-CALM	38	78.74 ± 10.125	5.71 ± 1.250	51.66 ± 11.252	51.32 ± 11.552	59.34 ± 13.581	103.03 ± 8.645	10.45 ± 3.438
CAU	39	75.44 ± 10.39	6.13 ± 0.695	49.31 ± 6.814	48.64 ± 4.934	55.00 ± 4.605	101.62 ± 9.35	10.05 ± 3.426
*t*		1.411	−1.806	1.105	1.315	1.869	0.687	0.506
*p*		0.162	0.076	0.273	0.194	0.068	0.494	0.614
After VR-CALM or CAU								
VR-CALM	38	100.47 ± 14.33	2.95 ± 1.845	44.16 ± 11.083	46.63 ± 9.824	55.82 ± 10.557	77.79 ± 11.548	8.74 ± 2.565
CAU	39	79.44 ± 6.703	5.49 ± 1.393	55.21 ± 3.806	50.21 ± 3.806	53.49 ± 6.328	99.77 ± 6.964	11.26 ± 4.494
*t*		8.216	−6.829	−5.819	−2.094	1.170	−10.082	−3.031
*p*		≤0.001	≤0.001	≤0.001	≤0.05	0.247	≤0.001	≤0.01

*Note.* FACT-B, Functional Assessment of Cancer Therapy-Breast cancer patient; DT, Distress Thermometer; SAS, The Self-Rating Anxiety Scale; SDS, The Self-Rating Depression Scale; CARS, Concerns About Recurrence Scale; PFS, Piper Fatigue Scale; PSQI, Pittsburgh Sleep Quality Index; SD, standard deviation. Data are presented as mean ± SD.

## Data Availability

Data supporting this research article are available from the corresponding author on reasonable request.

## Abbreviations

ACT: After CALM/CAU treatment
BCs: Breast cancer survivors
BCT: Before CALM/CAU treatment
CARS: Concerns About Recurrence Scale
CAU: Care as usual
CBT: Cognitive Behavioural Therapy
DT: Distress thermometer
EPCRC: European Palliative Care Research Collaborative
FACT-B: Functional Assessment of Cancer Therapy-Breast cancer patient
FCR: Fear of Cancer Recurrence
QOL: Quality of life
NCCN: National Comprehensive Cancer Network
PFS: Piper Fatigue Scale
PSQI: Pittsburgh Sleep Quality Index
RCT: Randomized controlled trial
SAS: The Self-Rating Anxiety Scale
SD: Standard deviation
SDS: The Self-Rating Depression Scale
VR-CALM: Managing Cancer and Living Meaningfully based on VR.
